# Impact of brewery sludge application on heavy metal build-up, translocation, growth and yield of bread wheat (*Triticum aestivum* L.) crop in Northern Ethiopia

**DOI:** 10.1016/j.heliyon.2024.e32559

**Published:** 2024-06-06

**Authors:** Wakjira Tesfahun Jebesa, Tessema Astatkie, Ambachew Zerfu, Hawi Deressa Kenea, Nezif Abamecha, Meresa Shumuye, Gezai Abera, Asmeret Kidane, Mignote Hirko, Fenta Assefa

**Affiliations:** a College of Agriculture and Veterinary Medicine, Jimma University, Jimma, Ethiopia; b Faculty of Agriculture, Dalhousie University, Truro, NS, Canada; c College of Agriculture and Natural Resource, Bonga University, Bonga, Ethiopia; d College of Agriculture and Natural Resource, Raya University, Maychew, Ethiopia; e International Livestock Research Institute, Addis Ababa, Ethiopia; f Department of Plant Sciences, College of Agriculture and Environmental Sciences, University of Gondar, Gondar, Ethiopia

**Keywords:** Heavy metals, Health assessment, Translocation, Brewery sludge

## Abstract

In a field study, the impact of different levels of brewery sludge (BS) enrichment on *Triticum aestivum* L. (wheat plants) was examined in terms of growth, yield, heavy metal absorption, and potential health risks linked to plant consumption. Using a randomized complete block design with seven treatments and three blocks, the study showed that applying up to 12 t ha^−1^ brewery sludge significantly improved all agronomic parameters (except harvest index) compared to control and mineral-fertilized soil. Heavy metal translocation was generally low, except for Cu and Pb. The sequence of heavy metal translocation was Cu > Pb > Cd > Ni > Zn > Mn > Cr from soil to spikes and Cu > Zn > Mn > Pb > Ni > Cd > Cr from soil to grain. Heavy metal loads were mostly higher in roots than in the above-ground crop parts. The target hazard quotient (THQ), hazard index (HI), and target cancer risk (TCR) within wheat grain remained within safe limits for all BS treatments. Consequently, consuming this wheat grain is considered safe regarding heavy metals. Thus, utilizing brewery sludge at 12 t ha^−1^ as a fertilizer for wheat production and as an alternative method for sludge disposal is plausible.

## Highlights

• Application of brewery sludge increased plant growth and yield parameters, ranging from 10 to 45 %.
• Applying brewery sludge over 12 t ha-1 decreased agronomic factors, raised heavy metal levels, and heightened health risks.
• The roots of wheat crops serve as a defense mechanism against heavy metal contamination.
• The heavy metal concentrations in wheat grain were below permissible limits.
• The translocation factor from root to grain in wheat plants was below 1, except for Cu and Pb.

## 1 Introduction

Brewery sludge (BS) is a waste product of the brewery industry that contains sufficient organic carbon-rich byproduct [1]. The soil enriched with sludge amendments can exhibit up to three times higher organic carbon levels than soil amended with inorganic fertilizers [2]. This establishes it as a favorable substance for enhancing soil quality. BS also contains macro- and micro-nutrients that are vital for plant development and can serve as a cheap source of organic matter for most agricultural soils [3]. Utilizing sludge in soil applications enhances nutrient availability, augments soil water retention capacity, refines soil structure and porosity and sustains organic matter [4]. Due to its enriched carbon composition, sludge is a viable amendment for soil degradation, exhibiting superior soil physical attributes including reduced bulk density, enhanced micro-aggregation, and improved hydraulic conductivity compared to inorganic fertilizers [5]. However, it is imperative to acknowledge that BS has the potential to harbor a notable concentration of heavy metals and pollutants [6]. With repetitive application, heavy metals have the propensity to accumulate both within the soil matrix and subsequently in the food chain, leading to phytotoxic effects and posing threats to human health and the environment [7]. These metals often manifest in carbonate or sulfide forms, frequently forming bonds with organic compounds. Their behavior and bioavailability within amended soils are contingent upon their specific speciation, along with the underlying physico-chemical properties of the sludge and the soil itself [8]. Consequently, the secure disposal of brewery sludge has emerged as a paramount global concern [9], because inadequate management of this waste substance bears the potential to introduce ecological contamination [10]. Currently, there are three methods of treating brewery sludge: incineration, landfilling, and land application as organic fertilizer or as soil conditioner [11]. However, the first two methods are not effective management practices because landfilling can contaminate groundwater, and incineration can cause environmental pollution.

The amendment of BS to the farming field continues to rise because it is an economically feasible and environmentally friendly management strategy [12]. Due to the elevated level of organic carbon (OC), cation exchange capacity, phosphorus, potassium, nitrogen, and other essential plant nutrients of brewery sludge, the fertility status of the soil will be improved, which results in enhanced productivity of crops [13]. Moreover, it may create favorable conditions for recycling nutrients and reduce the need for mineral fertilizers in farmlands [14]. The potential benefits of brewery sludge in enhancing soil fertility and crop productivity can be harnessed by solving the workable risks of heavy metals [10]. Heavy metals enter to human body system through water and food, which means excess heavy metal accumulation in the environment can have toxicological implications for humans. Moreover, these heavy metals are not easily biodegradable and can have far-reaching effects on the biological system [15].

The choice of the wheat crop for the present study stems from its significant global importance [16]. Wheat holds a pivotal position as a major crop contributing to income generation and bolstering food provision, particularly in sub-Saharan African nations [17]. Within Ethiopia, wheat stands as the third most substantial grain in terms of overall production (after maize and teff) and holds the fourth highest position in terms of land area coverage (surpassed by teff, maize, and sorghum) [18].

Limited scientific investigations have delved into the use of BS as an enhancer of crop production within the Ethiopian context. Among these studies, a notable work was done by Alayu and Leta [19] observed that applying brewery sludge at a rate of 0.96 t ha^−1^ increased maize yields by 11 % and 21.1 % compared to NPS fertilized and unfertilized plots, respectively. Similarly, Ahmed et al. [20] reported a significant increase in grain yield for haricot beans with the application of 12 t ha^−1^ of brewery sludge, enhancing yield by 53 % and 69.23 % compared to NP fertilized and control plots. Additionally, applying sludge at a rate of 160 Mg ha^−1^ improved certain physiological characteristics in crops, such as a 43 % increase in photosynthetic activity and a 60 % increase in stomatal conductivity compared to mineral fertilizer, resulting in better grain yield and nitrogen uptake in maize [21]. Furthermore, the grain weight of wheat improved by 80 % with the application of sludge at a rate of 100 t ha^−1^ compared to urea-fertilized plots [22], and barley grain yield significantly increased with the application of 5 t ha^−1^ of sludge [23], highlighting the benefits of brewery sludge amendments.

However, there is no study on brewery sludge application rate effects on wheat crop yield and productivity available in the literature. Therefore, this study was conducted to identify an appropriate brewery sludge application rate to increase wheat yield, and to determine where heavy metal accumulation is located in wheat tissues so that food chain contamination can be predicted more precisely. Also, heavy metal translocation, yield, and yield-related parameters of wheat are studied. The use of brewery sludge as a fertilizer in wheat crops is believed to have the potential to improve grain yield and other agronomic factors. The findings of the study can aid in determining the optimal amount of brewery sludge to apply, assessing the risk of heavy metal contamination, tracking the movement of heavy metals within wheat tissue, and establishing a foundation for governmental policies concerning the use of brewery sludge as a fertilizer in Ethiopian farmland.

## 2 Material and methods

### 2.1 Description of study area

**Fig. 1:**
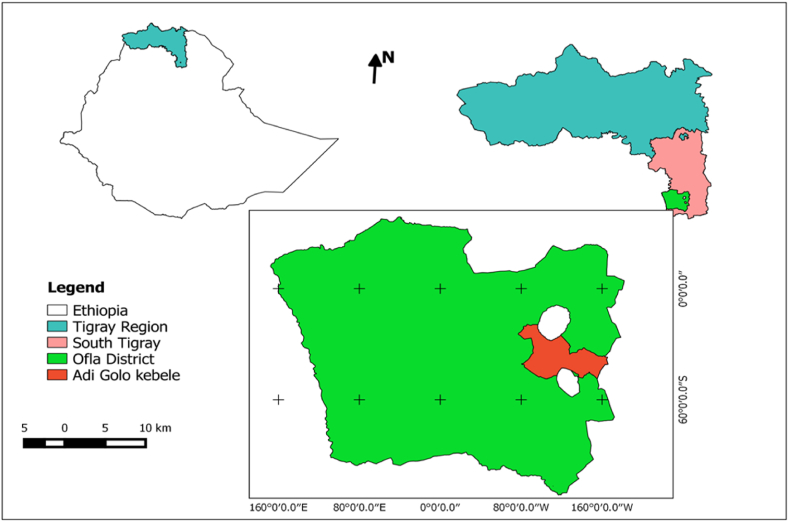
Map of the study area.

In the main cropping season of 2022, a study was conducted in the Ofla district of Southern Tigray, Ethiopia, located at 12°30'N and 39°31'E (Fig. 1). The district is characterized by a single rainy season, and its typical yearly precipitation is 986 mm. The average yearly temperature in this area is 15.3 °C, with a minimum average temperature of 5.4 °C and a maximum average temperature of 24.7 °C, as reported by Kidane et al. [24].

### 2.2 Treatments and experimental design

Twenty-one experimental units of 2 m × 3 m size were prepared for a Randomized Complete Block Design with three blocks and seven treatments. The treatments were Control (no amendments), five brewery sludge rates (3, 6, 9, 12, and 15 t ha^−1^), and one suggested rate (100 kg NPSBZn [17.8N- 35.7 P-7.75S- 0.1B- 2.2Zn] + 100 kg of Urea [46 %N]). Within each block, the distance between the seven plots was 0.5 m, and the distance between the blocks was 1 m. Air-dried brewery sludge was incorporated into the experimental field at the 5 rates one month before sowing. Full 100 kg of NPSBZn and half 32 kg of N as urea form was incorporated at the time of sowing and the half 32 kg of N was incorporated during tillering time as a side dressing to each plot. The selection of the “King Bird” wheat seed variety was driven by several factors including its remarkable yield potential, robust resilience to stem rust and various diseases, exceptional quality for bread-making, its pivotal role in upholding the livelihoods of farmers through the supply of food, livestock fodder, and income, as well as its notable contribution to overall caloric intake. The Kingbird bread wheat variety, sourced from Kulumsa Agricultural Research Center (KARC), was utilized in the experiments. It was seeded at a rate of 150 kg ha^−1^ in mid-July during the main cropping season.

### 2.3 Determination of soil physicochemical properties

Soil pH was determined by 1:2.5 in a soil-water ratio with a pH meter as described in Ref. [25]. Soil texture was determined according to the methods described in Ref. [26]. The soil organic matter content was determined using the potassium chromate (K_2_CrO_7_) digestion method as outlined in Ref. [27]. Available phosphorus, Potassium, and Total nitrogen were determined as described in Refs. [28,29], and [30], respectively. Soil cation exchange capacity (CEC) was determined from a saturated sample of ammonium acetate (NH_4_OAc) by using the micro Kjeldahl technique as outlined in Ref. [31]. The wheat and soil samples (ranging from 0.5 to 1.0 g for each type) underwent digestion by utilizing a combination of 9 mL of nitric acid (HNO_3_) and 3 mL of perchloric acid (HClO_4_) at a temperature of 200 °C for 2 h. The optimal measurement condition was established from a set of digestion techniques by identifying the procedure that achieved complete digestion of both samples while minimizing reagent consumption, digestion time, and temperature.

The validity and accuracy of the digestion and its subsequent analysis were performed by involving blank samples. The concentrations of Pb, Zn, Mn, Cr, Cu, Ni, and Cd were determined using an atomic absorption spectrophotometer (ASS). Standard solutions of metals (1000 mg L^−1^) were used to prepare working standards to establish calibration curves (0.5, 2.5, 5, 7.5, 8.5, and 10 mg L^−1^) in flame atomic absorption spectrometer. The concentration of elements in the blank was also determined by applying the same analytical procedure as with the sample. As can be seen in Supplementary Table S1, the coefficients of determination (R^2^) of the calibration curves varied between 0.9949 and 1.

### 2.4 Apparatuses and instruments

Samples of soil and wheat were placed within labeled zippered polyethylene (PE) bags, ensuring their protection from potential contamination, and promptly taken to the laboratory for subsequent analysis. The measurement of soil pH and conductivity was conducted using a Microprocessor-based PH-EC-TDS Meter (Model: 1615, ESICO). For the digestion of both wheat and soil samples, a Milestone Microwave (Model: STRAT D 134348, EVISA) was employed, while the drying process was carried out using a Drying Oven (Model: DHG-9123A). The processed samples were weighed using an Analytical balance (Model E11140, Switzerland). The quantification of various volumes of sample solutions, acid reagents, and metal standard solutions was achieved through the use of measuring cylinders, pipettes, and micropipettes (Merck KGaA, Darmstadt, Germany). The digestion process was performed under laboratory fume hood conditions, and the resultant digested samples were filtered using Whatman No. 42 filter paper. The determination of target metals in the studied wheat and soil samples was executed utilizing Flame Atomic Absorption Spectrophotometry (FAAS) (AA240FC, Australia) and the operating parameters presented in Supplementary Table S2.

### 2.5 Optimization of digestion procedures for soil and wheat samples

To determine the most suitable digestion procedure, a systematic approach was employed to optimize key parameters such as digestion time, reagent volume, and digestion temperature. This optimization process involved altering one parameter at a time while maintaining the others constant. The selection of optimal conditions was guided by factors such as the clarity of digested samples, minimal consumption of reagents, efficient digestion time, procedural simplicity, and the attainment of the lowest yet effective digestion temperature. Consequently, representative soil and wheat samples were individually taken, and controlled digestion procedures were carried out. This involved the systematic variation of one parameter while keeping the remaining factors unchanged. The optimization endeavors culminated in the identification of the optimal digestion condition for both soil and wheat samples. This condition entailed the use of 9 mL of HNO_3_ and 3 mL of HCl as acid reagents, a digestion time of 45 min, and specific pressure and temperature settings of 80W and 180 °C, respectively. These conditions were determined to be the most effective for achieving complete digestion of 0.5g samples of soil and wheat.

### 2.6 Method validation

The determination of the limit of quantification (LOQ) and the limit of detection (LOD) for all metals considered in this study is based on the response of the calibration reagent blank. The LOQ was established using the standard formula LOQ = 3 × SD, while the MDL was determined using the formula LOD = 10 × SD. These analytical values are comprehensively presented in Supplementary Table S3. The data analysis unveiled that the calculated LOD values ranged from 0.12 to 0.55 mg L^−1^, with corresponding LOQ values ranging between 0.4 and 1.81 mg L^−1^. These values illustrate that the utilized instrument demonstrated a heightened sensitivity for the conducted analysis.

#### 2.6.1 Precision and accuracy

The precision and accuracy of the method employed to investigate the presence of heavy metals within soil and wheat samples were rigorously validated through the application of the matrix spike recovery analysis technique. The resulting dataset is comprehensively presented in Supplementary Tables S4 and 5. Examination of the data reveals that the achieved recovery values ranged from 96.2 to 112 %, while the corresponding percentage relative standard deviations (% RSD) remained consistently below 4.59 % in all samples. It is noteworthy that the matrix spike recovery values obtained within this study align with the accepted benchmark of 80–120 % for robust recovery assessments [32]. The notably high percentage of recovery achieved in this study substantiates the method's accuracy and establishes its reliability in determining metal concentrations within both soil and wheat samples, which were the focal subjects of the analysis. Additionally, the attainment of low % RSD values (<15 %) underscores the method's precision, indicating its suitability for the meticulous analysis of heavy metal content.

### 2.7 Crop data collection and measurement

Wheat-yield -related parameters (spike length, plant height, and number of seed spike^−1^) were recorded from 10 randomly tagged plants from the central rows. The number of tillers and seeds per spike was determined by counting from the 1 m^2^ area of each plot. Data on grain and biomass yield were determined from the net plot (1.6 m × 2.6 m) by avoiding border effects. The sample of above-ground plants was subsequently dried in an oven for 72 h at 70 °C. Straw yield was determined as the difference between the total above-ground biomass and grain yield of the respective treatment. Thousand kernel weights were measured from the mass of counted thousand kernels using a sensitive electronic balance. The Harvest index was computed as the ratio of grain yield to the grain plus straw yield.

### 2.8 Human health risk assessment

#### 2.8.1 Daily intake of metals (DIM)

The daily intake of metals (DIM) was calculated using the formula DIM = (MC*DFI)/BW, as outlined by Khan et al. [33]. Here, MC represents the absorption of metals in wheat grain (mg kg^−1^), BW denotes the average body weight of a human, and DFI refers to the daily intake of wheat grain. A reference average body weight of 60.7 kg that was reported by Ref. [34] was utilized, along with an average daily intake of wheat grain of 0.088 kg person^−1^ day^−1^, as reported by Minot [35].

#### 2.8.2 Target hazard quotient (THQ)

The ratio of acquaintance heavy metals to the reference is used to express THQ. However, the reference dose differs depending on the trace element that is being evaluated.

$$\text{THQ}=\frac{\text{EF}\;x\;\text{FI}\;x\;\text{ED}\;x\;\text{MC}}{\text{RfD}\;x\;\text{AT}\;x\;\text{BW}}\;x\;0.001\text{,}$$

where EF is exposure frequency (for 365 days). The FI abbreviation refers to food ingestion, ED stands for exposure duration (which is assumed to be 60 years for adults), MC represents metal concentration in the food (measured in mg kg^−1^), RfD is an oral reference dose (measured in mg kg^−1^ day^−1^), AT stands for average exposure time for non-carcinogenic effects (computed as 365 days per year multiplied by the number of exposure years, assumed to be 60 years in this study), and BW denotes average adult body weight (which is assumed to be 60 kg). RfD estimates the amount of human daily exposure that is unlikely to cause adverse health effects during a person's lifetime.

#### 2.8.3 Hazard index (HI)

The abbreviation HI stands for the sum of the individual target hazard quotients of the elements evaluated for each food type. It calculated as

$$\text{HI}=\sum_{i=1}^{n}{THQ}_{i}$$

HI assumes that when a specific type of food is consumed, it may result in exposure to various potential toxic elements simultaneously. If HI is greater than 1, it indicates the possibility of adverse non-carcinogenic health effects [36].

#### *2.8.4* *Target cancer risk* (TCR)

TCR denotes the estimation of an individual's lifetime cancer risk based on their heavy metal ingestion. To calculate TCR, the USEPA Region III Risk-Based Concentration Table [37] is used, along with the following formula:

$$\text{TCR}=\frac{\text{Mc}\;x\;\text{IR}\;x\;\text{CPSo}\;x\;\text{EF}\;\text{xED}}{\text{BW}\;x\;\text{AT}}$$

where the variables Mc, IR (DFI), EF, ED, AT, and BW are explained above. CPSo stands for the oral cancer slope factor (measured in mg/kg/day). The values of CPSo for Pb, Cd, Cr, and Ni are listed in Table 1. As the corresponding values for other heavy metals were unavailable, their cancer risk could not be calculated.

**Table 1:** Reference doses (RfD) and Cancer Slope Factors (CSF) for different heavy metals.

Heavy metals	Oral reference dose *(mg/kg/day)*		Reference
Zn	0.3		
Ni	0.02		
Mn	0.14		[38]
Cu	0.04		
Pb	0.0035		[39]
Cr	0.003		
Cd	0.001		[40]
**Oral cancer slope factor *(mg/kg/day)***			
Cd	0.38		[41]
Ni	1.7		[38]
Pb	0.0085		[42]
Cr	0.5		[43]

### *2.9* *Translocation factor* (TF)

TF is a measure of wheat's capacity to transfer heavy metals from its roots to its shoot, spike, and grain tissues, as per the findings of Haddad et al. [44]. To calculate TF, the concentration of a particular heavy metal in the shoot, spike, and grain tissues (measured in mg kg^−1^) is divided by the concentration of the same heavy metal in the roots (measured in mg kg^−1^).

### 2.10 Statistical analysis

The statistical analyses were conducted using the General Linear Model (GLM) Procedure of SAS [45]. To verify the normal distribution and constant variance assumptions on the error terms for each response variable, a Normal Probability Plot of the residuals and a plot of the residuals vs. fitted values were created, respectively as explained in Montgomery [46]. The independence assumption was met through randomization of the treatments within each block. The treatment effect was significant at the 5 % level of significance for all response variables. Hence, multiple means comparison was performed using Fisher's LSD method at the 5 % level of significance to generate letter groupings [46].

## 3 Results

### 3.1 Physicochemical properties

As shown in Table 2, the site is characterized by clay textural classification with low TN, (0.05–0.15), moderate OC (1–1.19), high CEC (25–40 c^+^ mol), low salinity level, slightly alkaline pH, and high AVP and Exchangeable cations. The utilization of brewery sewage has triggered the introduction of a substantial amount of heavy metals into agricultural lands, subsequently leading to their uptake by crops intended for human consumption. These heavy metals, characterized by their non-degradability, persist in the soil over extended periods due to the absence of microbial or chemical degradation mechanisms. This persistence in the soil matrix has engendered a concerning ecological predicament, as the heavy metals infiltrate the food chain, disrupting the balance of ecosystems.

**Table 2:** Physicochemical properties of soil and brewery sludge.

Parameter	Agricultural soil	Brewery sludge
	Measured values	Measured values
Soil texture	Clay	–
pH	7.72	6.84
EC (mS/cm)	0.236	0.118
OC (%)	1.183	13.95
TN (%)	0.142	4.512
Av.P (ppm)	102.4	829.16
CEC (meq/100g of soil)	35.72	40.00
EX.K^+^ (ppm)	192.6	366.6
EX. Na^+^ (ppm)	99.5	456.5
EX.Mg^2+^(meq of 100g of soil)	22.08	10.72
EX.Ca^2+^(meq of 100g of soil)	23.84	64.96
Zn (mg/kg)	79	26.5
Cd (mg/kg)	0.36	1.24
Ni (mg/kg)	91	28.5
Pb (mg/kg)	0.48	0.65
Mn (mg/kg)	1907	0.58
Cu (mg/kg)	49	16.25
Cr (mg/kg)	68	0.51

Additionally, the presence of heavy metals exerts an adverse influence on the biodegradation capacity of organic pollutants, diminishing their degradability and compounding environmental pollution [47]. The implications of these heavy metals extend across the biosphere, as they can be ingested directly or absorbed by plants, thereby posing risks to both plants and the subsequent food chain. This phenomenon is accompanied by alterations in fundamental soil properties such as pH, color, porosity, and natural chemistry, consequently compromising overall soil quality. To comprehensively assess these effects, this study's findings were juxtaposed with recognized authorities (Table 3). The decision to draw upon these diverse authoritative sources was informed by their well-established scientific rigor, their orientation toward health and environmental safeguards, and their global regulatory recognition. Consequently, this study's outcome reveals that the concentrations of heavy metals within brewery sludge remain below the prescribed limits outlined by these esteemed standards. This suggests that the utilization of brewery sludge for agricultural purposes can be deemed safe, fostering soil fertility and crop productivity while mitigating potential environmental risks.

**Table 3:** Heavy metal limit values in sludge and soil intended for agricultural use (mg kg^−1^).

Type	Standard	Heavy metals						
**Sludge**		**Cd**	**Cu**	**Cr**	**Ni**	**Pb**	**Zn**	**Mn**
	Poland [48]	20	1000	500	300	750	2500	
	EU [49]	20–40	1000–1750	–	300–400	750–1200	2500–4000	–
	Chinese [50]	20	500	1000	200	1000	1000	–
	USA [51]	39	1500	–	420	300	2800	–
	South African a [52]	40	1500	1200	420	300	2800	280
	South African b [52]	85	4300	3000	420	840	7500	1225
**Soil**	EU [49]	3	100	100	50	100	450	–
	USEPA [37]	85	4300	3000	–	420	7500	–
	FAO/WHO [53]	0.3	100	100	50	100	99.4	–
	SEPA [54]	0.6	100	200	50	300	200	–
	CCME [55]	3	400	210	62	150	700	–

### 3.2 Yield and yield-related parameters

The application of up to 12 t ha^−1^ brewery sludge led to a significant (P < 0.01) increase in all vegetative and yield-related parameters of wheat, compared to the mineral fertilizer and the control treatments (Table 4). However, there was a significant decrease in wheat grain yield, plant height, spike length, and effective tiller at the 15 t ha^−1^ rate compared to the 12 t ha^−1^ rate (Table 4).

**Table 4:** Mean biomass yield [BY], grain yield [GY], thousand seed weight [TSW], straw yield [SY], Plant height [PH], spike length [SL], effective tiller [EL], total tiller [TT], and seed per spike [SS^−1^] obtained from different application rates of brewery sludge [BS].

BS (t ha^−1^)	BY (kg)	TSW (g)	GY (kg)	SY (kg)	PH (cm)	SL (cm)	ET	TT	SS^−1^
Control	5938^e^	31.53^e^	2977^d^	2961^c^	67.5^d^	6.66^d^	21.3^e^	30.0^d^	34.4^e^
NPSZnB	8958^b^	39.33^b^	4690^ab^	4268^ab^	85.9^b^	8.40^b^	43.3^b^	49.0^a^	49.5^a^
3	7865^d^	33.23^de^	3630^cd^	3865^ab^	82.4^c^	7.90^c^	33.7^d^	37.0^c^	42.6^d^
6	8071^cd^	34.63^d^	3885^bc^	4442^ab^	82.6^c^	7.96^c^	38.3^c^	44.0^b^	43.3^cd^
9	8750^bc^	36.67^c^	4000^bc^	4865^a^	83.6^bc^	8.27^bc^	39.2^c^	44.3^b^	46.3^bc^
12	10000^a^	41.47^a^	5297^a^	4979^a^	90.3^a^	9.27^a^	49.3^a^	51.3^a^	50.7^a^
15	9271^ab^	39.51^ab^	4292^bc^	4704^ab^	85.7^b^	8.53^b^	44.7^b^	48.3^a^	48.6^ab^

Within each column, means sharing the same letter are not significantly different at the 5 % level of significance.

### 3.3 Effects of brewery sludge on wheat tissues

As the application rate of brewery sludge increased, a highly significant (P < 0.01) increase in the concentration of all heavy metals was observed in different tissues of wheat as shown in Table 5. The highest concentrations of all heavy metals in all wheat tissues were obtained at the 15 t ha^−1^ brewery sludge application rate, while the control soil (0 t ha^−1^ of brewery sludge) and NPSBZn had the lowest concentrations. However, the concentrations of all heavy metals were within the normal range and did not reach phytotoxic levels, except for Zn (in roots), Cd (excluding grain), Cr (in roots) and Ni (in roots). Furthermore, the concentrations of heavy metals (except Cu), were higher in the roots than in the above-ground tissues. The pattern of heavy metal concentration in wheat tissues showed a linear increase with increasing brewery sludge application rate, as confirmed by the strong relationship of heavy metal content with BS application rate in the various parts of wheat tissues.

**Table 5:** Mean heavy metal concentrations (mg kg^−1^) in grains, spikes, shoots and roots of wheat obtained from the different application rates of brewery sludge [BS].

Metal	Tissue	BS (t ha^−1^)							Safe limit^a^	Phytotoxic Range^b^
		0	3	6	9	12	15	NPSBZn		
Zn	Grain	17.6^f^	21.0^d^	23.4^c^	24.2^c^	26.8^b^	32.1^a^	19.5^e^	60	100–500
	Spike	15.1^d^	16.6^d^	19.6^c^	20.3^c^	23.9^b^	26.7^a^	15.3^d^		
	Shoot	11.4^e^	13.8^d^	16.7^c^	17.0^c^	19.0^b^	22.8^a^	12.2^e^		
	Root	58.1^e^	63.0^d^	72.0^c^	83.5^b^	92.0^a^	93.7^a^	62.5^d^		
Pb	Grain	0.12^e^	0.13^e^	0.15^d^	0.18^c^	0.20^b^	0.24^a^	0.13^e^	5	30–300
	Spike	0.25^e^	0.54^d^	0.73^c^	0.80^bc^	0.85^b^	1.01^a^	0.32^e^		
	Shoot	0.14^f^	0.41^d^	0.46^c^	0.57^b^	0.86^a^	0.87^a^	0.25^e^		
	Root	0.47^d^	0.48^d^	0.49^d^	0.69^c^	0.74^b^	0.84^a^	0.42^e^		
Cd	Grain	0.02^ef^	0.03^de^	0.03^d^	0.05^c^	0.09^b^	0.12^a^	0.02^f^	0.3	5–30
	Spike	0.11^e^	0.20^d^	0.24^c^	0.24^c^	0.27^b^	0.34^a^	0.13^e^		
	Shoot	0.20^e^	0.23^cd^	0.24^cd^	0.25^c^	0.32^b^	0.39^a^	0.23^de^		
	Root	0.40^g^	0.51^e^	0.59^d^	0.66^c^	0.77^b^	0.81^a^	0.45^f^		
Cr	Grain	0.62^d^	0.66^d^	0.71^cd^	0.78^c^	1.01^b^	1.18^a^	0.52^e^	5	10–100
	Spike	0.35^e^	0.84^d^	1.00^d^	1.25^c^	2.04^b^	2.68^a^	0.45^e^		
	Shoot	0.44^e^	0.87^d^	0.88^d^	1.25^c^	2.15^b^	3.30^a^	0.31^e^		
	Root	15.6^e^	18.4^d^	22.1^c^	26.2^b^	34.2^a^	35.5^a^	13.0^f^		
Ni	Grain	10.7^e^	13.0^d^	13.4^cd^	14.1^c^	15.8^b^	17.0^a^	11.4^e^	20	40–246
	Spike	9.53^f^	16.0^d^	17.8^cd^	18.6^c^	21.8^b^	25.1^a^	13.9^e^		
	Shoot	10.4^e^	12.5^d^	13.6^c^	13.6^c^	15.4^b^	16.9^a^	15.0^b^		
	Root	33.7^g^	49.2^e^	54.7^d^	61.4^c^	66.1^b^	70.7^a^	39.1^f^		
Mn	Grain	119.9^d^	125.1^c^	126.0^c^	129.4^b^	136.2^a^	138.0^a^	118.9^d^	500	>400
	Spike	111.4^e^	114.5^e^	119.9^d^	127.2^c^	142.3^b^	148.1^a^	118.9^d^		
	Shoot	124.2^d^	132.2^c^	135.1^c^	147.1^b^	152.4^a^	155.5^a^	126.4^d^		
	Root	406.9^g^	410.5^f^	420.9^d^	434.6^c^	454.0^b^	470.8^a^	414.7^e^		
Cu	Grain	25.1^f^	27.0^e^	28.5^d^	30.0^c^	34.7^b^	36.3^a^	25.9^f^	40	20–100
	Spike	23.7^e^	28.5^d^	30.3^c^	31.8^b^	37.1^a^	37.9^a^	23.8^e^		
	Shoot	24.5^f^	29.2^d^	33.0^c^	33.1^b^	34.2^a^	34.9^a^	27.2^e^		
	Root	16.2^d^	17.0^d^	18.1^c^	18.6^c^	19.7^b^	20.9^a^	14.5^e^		

Within each row, means sharing the same letter are not significantly different at the 5 % level of significance.

a Kabata-Pendias [56].

b FAO/WHO [53].

### 3.4 Effects of brewery sludge on TF

The translocation factor (TF) values for heavy metals, except for Cu and Pb, were less than 1.0. The order of TF values was observed as follows: from roots to shoots, Cu > Pb > Cd > Ni > Zn > Mn > Cr; from roots to spikes, Cu > Pb > Cd > Ni > Mn > Zn > Cr; and from roots to grains, Cu > Zn > Mn > Pb > Ni > Cd > Cr. The tissues of wheat exposed to 15 t ha^−1^ of brewery sludge amendment had the highest TF values for most heavy metals, whereas the control and mineral fertilizer-treated plots had the lowest values, as shown in Table 6.

**Table 6:** Mean translocation factor (TF), from roots to shoots, spike and grain of heavy metals in wheat crop grown in soil with different application rates of brewery sludge [BS].

Metal	Factor	BS (t ha^−1^)						
		0	3	6	9	12	15	NPSBZn
Zn	TF grain	0.30^cd^	0.33^a^	0.32^a^	0.28^d^	0.29^d^	0.34^a^	0.31^bc^
	TF spike	0.25^abc^	0.26^abc^	0.27^ab^	0.24^c^	0.26^abc^	0.28^a^	0.24^bc^
	TF shoot	0.19^d^	0.21^bc^	0.23^ab^	0.20^cd^	0.20^cd^	0.24^a^	0.19^d^
Pb	TF grain	0.26^c^	0.27^bc^	0.32^a^	0.26^c^	0.27^bc^	0.28^bc^	0.31^ab^
	TF spike	0.53^d^	1.12^b^	1.49^a^	1.16^b^	1.14^b^	1.19^b^	0.75^c^
	TF shoot	0.29^f^	0.85^cd^	0.94^bc^	0.83^d^	1.16^a^	1.03^b^	0.60^e^
Cd	TF grain	0.07^d^	0.06^de^	0.06^de^	0.09^c^	0.11^b^	0.14^a^	0.05^e^
	TF spike	0.27^d^	0.39^abc^	0.41^ab^	0.36^bc^	0.35^c^	0.42^a^	0.28^d^
	TF shoot	0.50^a^	0.46^a^	0.41^b^	0.38^b^	0.41^b^	0.48^a^	0.48^a^
Cr	TF grain	0.04^a^	0.04^ab^	0.03^bc^	0.03^c^	0.03^c^	0.03^bc^	0.04^a^
	TF spike	0.02^e^	0.04^c^	0.04^c^	0.04^c^	0.05^b^	0.07^a^	0.03^d^
	TF shoot	0.02^e^	0.02^e^	0.04^d^	0.048^c^	0.06^b^	0.09^a^	0.02^e^
Ni	TF grain	0.31^a^	0.26^c^	0.24^d^	0.23^d^	0.23^d^	0.24^d^	0.29^b^
	TF spike	0.28c	0.32^abc^	0.32^ab^	0.30^bc^	0.33^ab^	0.35^a^	0.35^a^
	TF shoot	0.30^b^	0.25^c^	0.24^c^	0.22^d^	0.23^cd^	0.23^cd^	0.38^a^
Mn	TF grain	0.29^bc^	0.30^a^	0.299^abc^	0.297^bc^	0.30^ab^	0.29^c^	0.28^d^
	TF spike	0.27^d^	0.27^cd^	0.28^bc^	0.29^b^	0.31^a^	0.31^a^	0.28^bc^
	TF shoot	0.30^d^	0.32^bc^	0.32^c^	0.33^a^	0.33^a^	0.33^a^	0.30^d^
Cu	TF grain	1.55^c^	1.59^c^	1.58^c^	1.61^bc^	1.75^a^	1.75^a^	1.78^a^
	TF spike	1.46^d^	1.67^c^	1.67^c^	1.71^bc^	1.84^a^	1.81^ab^	1.80^ab^
	TF shoot	1.51^c^	1.72^b^	1.77^ab^	1.78^ab^	1.73^b^	1.67^b^	1.87^a^

Within each row, means sharing the same letter are not significantly different at the 5 % level of significance.

### 3.5 Effects on health assessments

#### 3.5.1 Effects of daily intake rate

The use of brewery sludge had a significant impact on the daily intake rates of the heavy metals studied. Among the wheat crop, the order of daily intake rates for the heavy metals was Mn > Cu > Zn > Ni > Cr > Pb > Cd (Table 7).

**Table 7:** Mean daily intake rate of heavy metals in wheat crop grown in soil amended with different application rates of brewery sludge [BS].

BS (t ha^−1^)	Daily intake rate (mg day^−1^)						
	Zn	Pb	Cd	Cr	Ni	Mn	Cu
0	2.1x10^−3f^	1.5x10^−5e^	3.4x10^−6ef^	7.70x10^−5d^	1.30x10^−3e^	1.47x10^−2d^	3.00x10^−3f^
3	2.5x10^−3d^	1.6x10^−5e^	4.0x10^−6ed^	8.00x10^−5d^	1.60x10^−3d^	1.54x10^−2c^	3.30x10^−3e^
6	2.8x10^−3c^	1.9x10^−3d^	4.7x10^−6d^	8.70x10^−5cd^	1.65x10^−3cd^	1.55x10^−2c^	3.51x10^−3d^
9	2.9x10^−3c^	2.2x10^−5c^	7.3x10^−6c^	9.60x10^−5c^	1.74x10^−3c^	1.59x10^−2b^	3.69x10^−3c^
12	3.2x10^−3b^	2.5x10^−5b^	1.1x10^−5b^	1.25x10^−4b^	1.94x10^−3b^	1.67x10^−2a^	4.20x10^−3b^
15	3.9x10^−3a^	2.9x10^−5a^	1.4x10^−5a^	1.45x10^−4a^	2.00x10^−3a^	1.70x10^−2a^	4.40x10^−3a^
NPSZnB	2.4x10^−3e^	1.6x10^−3c^	2.9x10^−6f^	6.40x10^−5e^	1.40x10^−3e^	1.46x10^−2d^	3.10x10^−3f^

Within each column, means sharing the same letter are not significantly different at the 5 % level of significance.

#### *3.5.2* *Effects on target cancer risk* (TCR)

The TCR values of Cd, Cr, Pb, and Ni were observed to fall within the range of 1.1x10-6 to 5.62x10-6, 3.2x10^−5^ to 7.2x10^−5^, 1.29x10^−7^ to 2.15x10^−7^, and 2.2x10^−5^ to 3.5x10^−5^, respectively. According to the USEPA [37] guidelines, a cancer risk lower than 1x10^−6^ is considered negligible, while a value greater than 1x10^−4^ is deemed unacceptably high. The range between 1x10^−4^ and 1x10^−6^ is deemed acceptable. The study results showed that the carcinogenic risk for Pb was lower than the negligible level, whereas the other heavy metals, namely Cd, Cr, and Ni, fell within the acceptable range (Table 8).

**Table 8:** Mean target cancer risk (TCR) of heavy metals in wheat grain grown with different application rates of brewery sludge [BS].

BS (t ha^−1^)	Target Cancer Risk			
	Cd	Cr	Pb	Ni
0	1.29x10^−6ef^	3.8x10^−5d^	1.29x10^−7e^	2.2x10^−5e^
3	1.53x10^−6de^	4.0x10^−5d^	1.40x10^−7e^	2.7x10^−5d^
6	1.81x10^−6d^	4.3x10^−5cd^	1.66x10^−7d^	2.8x10^−5cd^
9	2.79x10^−6c^	4.8x10^−5c^	1.88x10^−7c^	2.9x10^−5c^
12	4.32x10^−6b^	6.2x10^−5b^	2.16x10^−7b^	3.3x10^−5b^
15	5.62x10^−6a^	7.2x10^−5a^	2.15x10^−7a^	3.5x10^−5a^
NPSZnB	1.10x10^−6f^	3.2x10^−5e^	1.39x10^−7e^	2.3x10^−5e^

Within each column, means sharing the same letter are not significantly different at the 5 % level of significance.

#### *3.5.3* *Effects on target hazard quotient* (THQ)

All heavy metals evaluated in this study had THQ values below the critical level of 1.00. The highest THQ value was observed for Mn with an application rate of 12 t ha^−1^ of BS, followed by decreasing values for Cu, Ni, Cr, Zn, Cd, and Pb at the highest BS application rate. These findings suggest that there is no significant potential for non-carcinogenic health risks associated with the consumption of wheat grain produced from soil treated with BS, as per the results presented in Table 9.

**Table 9:** Mean Target Hazard quotients and hazard index (HI) of the heavy metals in wheat grown with different application rates of brewery sludge [BS].

BS (t ha^−1^)	Target Hazard quotient (mg day^−1^)							HI
	Zn	Pb	Cd	Cr	Ni	Mn	Cu	
0	7.20x10^−3f^	1.50x10^−3e^	3.40x10^−3ef^	2.50x10^−2d^	6.5x10^−2e^	1.0x10^−1d^	7.7x10^−2f^	0.28^f^
3	8.60x10^−3d^	1.64x10^−3e^	4.00x10^−3ed^	2.69x10^−2d^	8.0x10^−2d^	1.1x10^−1c^	8.3x10^−2e^	0.31^e^
6	9.60x10^−3c^	1.95x10^−3d^	4.70x10^−3d^	2.90x10^−2cd^	8.2x10^−2cd^	1.1x10^−1c^	8.7x10^−2d^	0.32^d^
9	9.90x10^−3c^	2.20x10^−3c^	7.35x10^−3c^	3.20x10^−2c^	8.7x10^−2c^	1.1x10^−1b^	9.2x10^−2c^	0.34^c^
12	1.09x10^−2b^	2.54x10^−3b^	1.10x10^−2b^	4.17x10^−2b^	9.7x10^−2b^	1.2x10^−1a^	1.0x10^−1b^	0.39^b^
15	1.31x10^−2a^	2.95x10^−3a^	1.47x10^−2a^	4.84x10^−2a^	1.0x10^−1a^	1.2x10^−1a^	1.1x10^−1a^	0.41^a^
NPSZnB	8.00x10^−3e^	1.64x10^−3e^	2.90x10^−3f^	2.10x10^−2e^	7.0x10^−2e^	1.0x10^−1d^	7.9x10^−2f^	0.28^f^

Within each column, means sharing the same letter are not significantly different at the 5 % level of significance.

#### *3.5.4* *Effects on hazard index* (HI)

The heavy metal HI values observed in the study were below the established threshold value. The highest HI value (0.41) was detected in the soil amended with 15 t ha^−1^ of BS, while the lowest value (0.28) was measured in the control and mineral fertilizer-treated plots. Additionally, the results indicated that as the BS application rate increased, the HI values also increased (Table 9).

## 4 Discussion

The increase in plant height may be attributed to the presence of macro- and micro-nutrients that are released from the decomposition of brewery sludge. These nutrients are believed to enhance the activity of cell division and cell expansion, which can lead to an increase in plant height. 10.13039/100014337Furthermore, the positive outcome could potentially be attributed to the improved soil porosity, water-holding capacity, increased stability of aggregates, diminished soil evaporation, lowered bulk density, and the introduction of supplementary carbon through the incorporation of soil amendments [57]. Supporting this assertion, earlier research has shown that applying sludge increased the plant height of maize [58], and ryegrass [59]. Furthermore, within a rice-wheat cropping system, the application of sludge at a rate of 30 Mg ha^−1^ led to a substantial increase in plant height compared to the usage of recommended mineral fertilizer [5].

The addition of brewery sludge as an amendment resulted in a significant increase in the number of fertile and total tillers, surpassing those of the control and mineral fertilizer treatments. This increase may be attributed to the high organic carbon content of the sludge, which releases macronutrients, especially soil NPK, improving nutrient availability in the soil and promoting plant growth and development. The results align with those of Boudjabi et al. [22] who indicated a significant increase (47.58 %) in wheat tiller numbers when applying 100 t ha^−1^ of sludge, as opposed to using mineral fertilizer. The number of grains per spike and the number of tillers positively affect this yield component. It is known that this parameter of fertility closely depends on the amount of phosphorus in the soil. Indeed, the sludge used in this study is rich in this element and its enduring mineralization provides a significant amount of this mineral for plants and subsequently ensures a gradual growth in the number of grains per spike and fertile tillers [60].

The positive effect of brewery sludge on grain yield may be attributed to the increase in nutrient availability and improvement in physicochemical properties of the soil, including lower soil pH and bulk density, enhanced organic matter, and improved porosity, leading to improved yield components of wheat. Amendments using brewery sludge have been shown to increase the biomass of crops such as sorghum, maize, and wheat [19,61,62]. Moreover, Eid et al. [9] mentioned that the biomass and the growth parameters (except the shoot/root ratio) of spinach showed a positive response to sewage sludge applications of up to 40 g kg^−1^ compared to the control soil. Furthermore, the application of sludge may have increased the photosynthesis capacity of the crop by improving the radiation efficiency of CO_2_ assimilation and coefficient photochemical quenching and transpiration [63]. In our study, all agronomic traits decreased in response to 15 t ha^−1^ of brewery sludge. A similar finding was reported on spinach plants [9]. A high sludge content was reported to suppress plant growth hormones (auxin and gibberellin), which are responsible for the growth and development of plants [64]. The heavy metals entering the protoplasm may cause a reduction in plant growth at high brewery sludge concentrations, resulting in the loss of intermediary metabolites, which are important for the growth and development of plants [65]. The higher build-up of heavy metals in plants causes a reduced photosynthetic rate, a decline in transpiration rate and chlorophyll pigments, disturbed photochemical light quenching, increased lipid peroxidation, proline and protein contents and stunted growth, and lowering of yield [66]. Heavy metals at high concentrations in a growth media can act as stressors, initiating physiological constraints that reduce plant vigor and limit plant growth, development, and biomass [64]. The production of reactive oxygen species and free radicals in plants is stimulated by heavy metal stress [67]. These free radicals can disrupt normal metabolism through oxidative damage to plant cellular components because these species have a strong oxidizing property and can attack all types of biomolecules [66].

The Harvest index was not significantly affected by brewery sludge application. This indicates that plants supply a large amount of photosynthates assimilation to reproductive organs to obtain a higher harvest index as compared to vegetative growth. However, from each increment of brewery sludge amendment, it looks like plants are shifting the photosynthates products from the reproductive part to heal impairment that arises from a large amount of trace metal concentration and therefore a significant increment in harvest index was not recorded among brewery sludge applications. These results agree with those of Singh and Agrawal [67] who found a non-significant difference in the harvest index of *Vigna radiata* among treatments.

The current study's findings reveal that the roots exhibited the highest concentrations of most heavy metals, contrasting with the shoot tissues (including spike, shoot, and grain). This trend aligns with a previous study by Eid et al. [9], suggesting a more pronounced accumulation of heavy metals in roots compared to in shoots, particularly when sludge applications are employed. This phenomenon can be attributed to specific root mechanisms facilitating heavy metal sequestration. For instance, low molecular weight proteins like phytochelatins and phytosiderophores, acting as heavy metal chelators, may contribute to diminishing heavy metal levels in shoots and grains [68]. These results imply the existence of a plant defence mechanism, known as the “root barrier” [69], which concentrates heavy metal build-up in the roots, thus restricting their movement through the food chain. This process prevents excessive and toxic accumulation of heavy metals in the plant's edible portions [70]. Plants employing this strategy primarily store absorbed heavy metals within root cells, either by chelating them in the cytoplasm or sequestering them in vacuoles [71]. The significant accumulation of heavy metals in roots could be attributed to their complexation with sulfhydryl groups, potentially leading to reduced translocation to the shoots [72]. Furthermore, the elevated concentrations of heavy metals in roots compared to shoots may be due to roots being the initial point of contact with heavy metals, consequently resulting in higher accumulation within root tissues [73]. To mitigate the adverse effects of heavy metal translocation, an effective approach involves prevention. By avoiding heavy metal movement into the shoot and edible portions of the wheat crop, negative impacts can be minimized [74]. Achieving this goal when plants are exposed to elevated heavy metal levels involves inducing lignin synthesis to thicken the root cell wall [75]. Additionally, the root apoplast pathway acts as a barrier limiting heavy metal entry into the symplastic pathway, and lipid peroxidation is another mechanism constraining heavy metal movement [76]. The findings of our study are consistent with those of [9,73] demonstrating the propensity for heavy metals to accumulate in roots rather than in shoots, underscoring the essential role of roots in safeguarding edible plant parts from heavy metal contamination.

The translocation of heavy metals from roots to shoots is governed by multiple factors, including physiological characteristics, crop type, and water transport mechanisms. In this study, most of the studied heavy metals had a TF value of less than 1, indicating that roots act as a barrier to translocation and protect the edible portion from toxicity. Antoniadis et al. [77] reported that heavy metal translocation decreases significantly as the metals move away from the roots, suggesting that roots accumulate more heavy metals than aboveground parts. Additionally, Sharma et al. [78] concluded that lower molecular complexes and ions are more mobile than other forms. Although roots may absorb more heavy metals, their distribution and translocation to aerial crop parts are restricted by high molecular weight and complexes. Kenaf plants retain 85–96 % of the total heavy metals present in the plants [79], *Thlaspi praecox Wulf*. can retain 80 % of heavy metal loads in the root [80], *Brassica juncea* can accumulate over 95 % of the total heavy metals in the roots [81], and Eid et al. [9] found significant retention of heavy metals in wheat crop and *Spinacia oleracea*.

According to Titilawo et al. [82], carcinogenic risk is considered high if it exceeds 4.0, low if it falls between 0.1 and 1, and negligible if it is below 0.1. The present study's values were below 0.1, indicating that consuming wheat crops grown in soils treated with BS has no adverse health effects. Li et al. [83] defined TCR between 1x10^−6^ and 1x10^−5^ as low risk, between 1x10^−5^ and 5x10^−4^ as medium risk, and between 5x10^−4^ and 1x10^−3^ as high risk. The TCR values obtained in this study were all below 1x10^−6^, which indicates a very low risk of developing cancer in individuals exposed to the metallic elements investigated.

## 5 Conclusion

The study aimed to assess the influence of various rates of brewery sludge application on wheat (*Triticum aestivum* L.) growth, yield, heavy metal absorption, translocation, and potential human health implications tied to wheat consumption. The findings highlighted the positive effects of brewery sludge on wheat growth and yield parameters. Heavy metal accumulation in wheat components (roots, shoots, spikes, and grain) remained within safe levels, with roots showing higher metal accumulation. Except for Pb and Cu, the translocation factor was below 1 for all metals. The study demonstrated that the estimated daily intake of these metals through wheat consumption fell below the permissible threshold (0.088). Both total health quotient (THQ) and health index (HI) related to metal intake were below 1, suggesting no significant health risks. Brewery sludge application's suitability as fertilizer depends on soil characteristics, including texture, moisture, redox potential, cation exchange capacity, pH, and plant species. The study's scope was limited to direct impacts on wheat, warranting further investigation into residual effects on yield, heavy metal content, and soil fertility of other cereals. A long-term field experiment, with varying soil types and sludge application rates, is needed to explore heavy metal accumulation and bioavailability. While the study focused on specific heavy metals, expanding to include others like Fe, Mo, Ar, and Hg could offer comprehensive insights into soil, crop, and human health. Notably, the research omitted analysing heavy metal concentrations in plants and their nutritional composition. More comprehensive studies are needed to understand heavy metal contamination's effects on plant nutritional aspects. Based on the study, an application rate of 12 t ha^−1^ appears to be optimal to mitigate environmental and health risks for animals and humans. These data provide a foundational understanding, suggesting a need for large-scale, multi-year investigations for robust and generalizable conclusions. In conclusion, the impact of brewery sludge on wheat cultivation is favorable in controlled conditions, yet further exploration is essential to validate these findings under broader agricultural contexts.

## CRediT authorship contribution statement

**Wakjira Tesfahun:** Writing – review & editing, Writing – original draft, Visualization, Validation, Supervision, Software, Resources, Project administration, Methodology, Investigation, Funding acquisition, Formal analysis, Data curation, Conceptualization. **Tessema Astatkie:** Writing – review & editing, Visualization, Software, Methodology, Formal analysis, Data curation. **Ambachew Zerfu:** Writing – review & editing, Validation, Supervision, Methodology, Investigation, Conceptualization. **Hawi Deressa Kenea:** Writing – review & editing, Validation. **Nezif Abamecha:** Writing – review & editing, Data curation. **Meresa Shumuye:** Writing – review & editing, Validation, Supervision, Project administration, Funding acquisition, Conceptualization. **Gezai Abera:** Writing – review & editing, Supervision, Project administration, Funding acquisition. **Asmeret Kidane:** Writing – review & editing, Validation, Supervision, Project administration, Funding acquisition. **Mignote Hirko:** Writing – review & editing. **Fenta Assefa:** Writing – review & editing.

## Declaration of competing interest

The authors declare that they have no known competing financial interests or personal relationships that could have appeared to influence the work reported in this paper.

## Abbreviations

AT: Average exposure Time
BS: Brewery sludge
BW: Body weight
CCME: Canadian Council of the Ministers of the Environment
CPSO: Oral cancer slope factor
DFI: Daily food ingestion
DIM: Daily intake of metals
ED: Exposure duration
EF: Exposure frequency
EU: European Union
FAAS: Flame Atomic Absorption Spectrophotometry
FAO: Food Association Organization
FI: Food ingestion
HI: Hazard index
LOD: Limit of detection
LOQ: Limit of quantification
LSD: Least significant difference
MC: Metal concentration
MDL-: Method of detection limit
RFD: Oral reference doze
RSD: Relative standard detection
SAS: Statistical Analysis Software
SD: Standard deviation
SEPA: State Environmental Protection Administration
TCR: Target Cancer risk
TF: Translocation factor
THQ: Target hazard quotient
USEPA: United States Environmental Protection Agency
WHO: World Health Organization
