# Supplementary material for: Impact of brewery sludge application on heavy metal build-up, translocation, growth and yield of bread wheat (Triticum aestivum L.) crop in Northern Ethiopia
Source: Heliyon. 2024 Jun 6;10(11):e32559. doi: 10.1016/j.heliyon.2024.e32559 (PMC11219491; doi:10.1016/j.heliyon.2024.e32559)
Supplement: Multimedia component 1 [file mmc1.docx]

**Table S1**

Working standard concentrations, regression equation, and correlation coefficient for determination of metals in wheat and soil samples.

| **Metal analyzed** | **Concentration (mgL^-1^)** | **Regression**  **Equation** | **R^2^** |
| --- | --- | --- | --- |
| Pb | 0.5, 2.5, 5.0, 7.5, 8.5, 10 | y=0.00398x-0.0 0025 | 0.99889 |
| Cr | 0.5, 2.5, 5.0, 7.5, 8.5, 10 | y= 0.00639x +0.0228 | 0.99663 |
| Cu | 0.5, 2.5, 5.0, 7.5, 8.5, 10 | y=0.0106x+0.000058 | 1 |
| Cd | 0.5, 2.5, 5.0, 7.5, 8.5, 10 | y= 0.01286x+0.0052 | 0.99785 |
| Zn | 0.5, 2.5, 5.0, 7.5, 8.5, 10 | y= 0.0007x+ 0.0002 | 0.9949 |
| Mn | 0.5, 2.5, 5.0, 7.5, 8.5, 10 | y= 0.00936x+0.0852 | 0.98843 |
| Ni | 0.5, 2.5, 5.0, 7.5, 8.5, 10 | y= 0.00802x+0.0027 | 1.0000 |

Standard solutions of metals (1000 mgL^-1^) were used to prepare working standards to establish calibration curves (0.5, 2.5, 5, 7.5, 8.5, and 10 mgL^-1^) in flame atomic absorption spectrometer. The concentration of elements in the blank was also determined by applying the same analytical procedure as with the sample. As can be seen in Table S3, correlation coefficients (R^2^) of the calibration curves were varied between 0.9949 and 1.

**Table S**2

Operating parameters for FAAS.

| **Element** | **Cd** | **Cu** | **Cr** | **Mn** | **Ni** | **Pb** | **Zn** |
| --- | --- | --- | --- | --- | --- | --- | --- |
| Lamp current (mA) | 4 | 4 | 7 | 5 | 4 | 5 | 5 |
| Fuel | C_2_H_2_ | C_2_H_2_ | C_2_H_2_ | C_2_H_2_ | C_2_H_2_ | C_2_H_2_ | C_2_H_2_ |
| Support | Air | Air | N_2_O | Air | Air | N_2_O | Air |
| Wavelength (nm) | 228.8 | 324.7 | 357.9 | 275.9 | 232.0 | 217.0 | 213.9 |
| Slit width (nm) | 0.5 | 0.5 | 0.2 | 0.2 | 0.2 | 1.0 | 1.0 |

**Table S3**

Recovery results for Validation of the Optimized Procedure (mean, mgkg^-1^, n = 3) for wheat and soil samples.

| Heavy metals | Wheat samples |  | Soil samples | |
| --- | --- | --- | --- | --- |
|  | LOD | LOQ | LOD | LOQ |
| Pb | 0.5 | 1.65 | 0.12 | 0.4 |
| Cr | 0.27 | 0.89 | 0.25 | 0.83 |
| Cu | 0.55 | 1.81 | 0.14 | 0.46 |
| Cd | 0.29 | 0.96 | 0.16 | 0.53 |
| Zn | 0.45 | 1.48 | 0.45 | 1.49 |
| Mn | 0.22 | 0.73 | 0.49 | 1.63 |
| Ni | 0.25 | 0.82 | 0.41 | 1.36 |

LOD: Limit of Detection

LOQ: Limit of Quantification

**Table S4**

Percentage recovery values of the method used for wheat digestion (Mean±SD, n = 3).

| **Heavy metals** | **Amount spiked (ppm)** | **Concentration after spiking**  **(ppm)** | **Concentration before spiking**  **(ppm)** | **% Recovery** | **% RSD** |
| --- | --- | --- | --- | --- | --- |
| Zn | 5 | 5.78±0.08 | 0.28±0.05 | 110 | 2.15 |
| Cd | 5 | 5.85±0.15 | 0.25±0.06 | 112 | 2.09 |
| Ni | 5 | 5.7±0.12 | 0.25±0.01 | 109 | 1.25 |
| Pb | 5 | 5.68±0.08 | 0.28±0.02 | 108 | 2.59 |
| Mn | 5 | 5.2±0.05 | 0.22±0.01 | 99.6 | 2.45 |
| Cu | 5 | 5.15±0.11 | 0.31±0.04 | 96.8 | 1.96 |
| Cr | 5 | 5.1±0.09 | 0.15±0.01 | 99 | 2.40 |

**Table S5**

Percentage recovery values of the method used for soil samples digestion (Mean ±SD, n = 3).

| **Heavy metals** | **Amount spiked (ppm)** | **Concentration after spiking**  **(ppm)** | **Concentration before spiking**  **(ppm)** | **% Recovery** | **% RSD** |
| --- | --- | --- | --- | --- | --- |
| Zn | 5 | 5.24±0.14 | 0.39±0.02 | 97 | 3.41 |
| Cd | 5 | 5.56±0.08 | 0.75±0.01 | 96.2 | 4.25 |
| Ni | 5 | 6.15±0.16 | 0.92±0.02 | 104.6 | 3.28 |
| Pb | 5 | 6.25±0.11 | 1.05±0.05 | 104 | 2.99 |
| Mn | 5 | 6.54±0.13 | 1.08±0.07 | 109.2 | 2.72 |
| Cu | 5 | 5.63±0.09 | 0.52±0.01 | 102.2 | 4.59 |
| Cr | 5 | 6.28±0.12 | 0.88±0.03 | 108 | 3.06 |
